# Distinctive features of the central synaptic organization of *Drosophila* larval proprioceptors

**DOI:** 10.3389/fncir.2023.1223334

**Published:** 2023-07-26

**Authors:** Marie R. Greaney, Chris C. Wreden, Ellie S. Heckscher

**Affiliations:** ^1^Committee on Neurobiology, The University of Chicago, Chicago, IL, United States; ^2^Department of Molecular Genetics and Cell Biology, The University of Chicago, Chicago, IL, United States; ^3^Institute for Neuroscience, The University of Chicago, Chicago, IL, United States

**Keywords:** proprioception, somatosensation, synapses, connectome, nociception

## Abstract

Proprioceptive feedback is critically needed for locomotor control, but how this information is incorporated into central proprioceptive processing circuits remains poorly understood. Circuit organization emerges from the spatial distribution of synaptic connections between neurons. This distribution is difficult to discern in model systems where only a few cells can be probed simultaneously. Therefore, we turned to a relatively simple and accessible nervous system to ask: how are proprioceptors’ input and output synapses organized in space, and what principles underlie this organization? Using the *Drosophila* larval connectome, we generated a map of the input and output synapses of 34 proprioceptors in several adjacent body segments (5–6 left-right pairs per segment). We characterized the spatial organization of these synapses, and compared this organization to that of other somatosensory neurons’ synapses. We found three distinguishing features of larval proprioceptor synapses: (1) Generally, individual proprioceptor types display segmental somatotopy. (2) Proprioceptor output synapses both converge and diverge in space; they are organized into six spatial domains, each containing a unique set of one or more proprioceptors. Proprioceptors form output synapses along the proximal axonal entry pathway into the neuropil. (3) Proprioceptors receive few inhibitory input synapses. Further, we find that these three features do not apply to other larval somatosensory neurons. Thus, we have generated the most comprehensive map to date of how proprioceptor synapses are centrally organized. This map documents previously undescribed features of proprioceptors, raises questions about underlying developmental mechanisms, and has implications for downstream proprioceptive processing circuits.

## Introduction

Locomotion critically requires feedback from multiple morphological types of proprioceptors that sense different features of the body’s posture and movement (Rossignol et al., 2006; Mendes et al., 2013; Akay et al., 2014; Bidaye et al., 2017). While loss of one type or another separately has minimal effect, the loss of multiple types produces severe locomotor deficits (Hughes and Thomas, 2007; Akay et al., 2014; Santuz et al., 2019), demonstrating that information from multiple proprioceptive neuron types is combined centrally for locomotor control. Despite their importance for locomotion, relative to other sensory neurons, little is understood about how proprioceptive neurons are anatomically organized once they project into the central nervous system (CNS). The question of sensory mapping has long been fundamental to our understanding of sensory processing (Hubel and Wiesel, 1962; Knudsen et al., 1987; Giessel and Datta, 2014; Hildebrandt, 2014); but we know little about whether, or what form of, topographical maps exist for this essential sense.

Decades of work on proprioceptive circuits has built up a general picture of how proprioceptive afferents project into the CNS. These studies have largely relied on single-neuron tracing and electrophysiological approaches (Scheibel and Scheibel, 1969; Burrows, 1975; Brown and Fyffe, 1978, 1979; Hustert, 1982; Conradi et al., 1983; Watson and Bazzaz, 2001) and recently have been complemented by genetic techniques, especially in mice and flies (Smith and Shepherd, 1996; Sürmeli et al., 2011; Niu et al., 2013; Agrawal et al., 2020). Certain anatomical principles regarding afferent projections hold across animal systems: first, like afferents of other sensory systems, proprioceptors receive synapses from inhibitory neurons (Pearson and Goodman, 1981; Burrows and Matheson, 1994; Clarac and Cattaert, 1996; Eggers et al., 2007; Wilson, 2013; Fink et al., 2014; Chen et al., 2021). Second, proprioceptive afferents are largely segregated from exteroceptive afferents (Brown, 1981; Murphey et al., 1985; Pflüger et al., 1988). Third, afferent projections diverge based on proprioceptor type (Brown, 1981; Murphey et al., 1985). Fourth, individual proprioceptor afferents often project to multiple locations (Burrows and Pflüger, 1988; Jankowska, 2008). Lastly, in addition to diverging, proprioceptors can converge in their connectivity, providing common input to downstream neurons that receive synapses from multiple proprioceptor types (Lundberg, 1979; Jankowska, 1992; Gebehart et al., 2021).

Beyond these principles, however, prior mapping efforts have revealed a complicated spatial organization of proprioceptor afferents (Scheibel and Scheibel, 1969; Edgley and Jankowska, 1987; Pflüger et al., 1988; Prasad and Weiner, 2011; Wu et al., 2021) that does not resemble the structured, feature-based mapping seen in other sensory systems. In part because of this complexity, we have only a partial understanding of afferent anatomy, even in what is arguably the best characterized of any proprioceptive CNS region, the locust thoracic ganglia (Tyrer and Gregory, 1982; Pflüger et al., 1988; Bidaye et al., 2017). Two limitations have impeded the anatomical mapping of proprioceptors into the CNS. First, only a single cell or cell type is typically mapped at a time, providing a piecemeal rather than integrative representation of how proprioceptive central axonal projections are anatomically organized (Fyffe and Light, 1984; Smith and Shepherd, 1996; Bidaye et al., 2017). This is particularly problematic for understanding the extent and form of divergence and convergence. Second, most mapping studies do not characterize the anatomical synapses, which are the actual sites of chemical information transfer; this gap may obscure organizational principles that would emerge at the synaptic level of anatomy. We could therefore gain substantially from mapping all proprioceptors simultaneously at synaptic resolution.

This study aims to map the input and output synapses of all types of *Drosophila* larval proprioceptors. Comprehensive mapping at the synaptic level of detail is possible in this system thanks to the availability of an electron micrographic dataset and connectome (Saalfeld et al., 2009; Ohyama et al., 2015; Schneider-Mizell et al., 2016). Moreover, because larvae are a relatively transparent genetic model, they are amenable to non-invasive, light-based techniques for probing circuit function, such as calcium imaging and optogenetic manipulation (Jovanic et al., 2016; He et al., 2019; Vaadia et al., 2019; Tadres and Louis, 2020). Non-invasive techniques are especially important for studying how the sensing of self-movement by proprioceptors tunes locomotive behavior. Therefore, a better understanding of proprioceptors in *Drosophila* larvae will ultimately complement and enable developmental and functional studies of proprioception, locomotion, and the underlying circuit-level mechanisms. Like other animals, *Drosophila* larvae have multiple types of proprioceptors which sense various features of movement (Grueber et al., 2002; Suslak and Jarman, 2015; He et al., 2019; Vaadia et al., 2019); these proprioceptors are critically needed for larval locomotion (Hughes and Thomas, 2007; Song et al., 2007). Also as in other systems, functional (Hughes and Thomas, 2007) and anatomical (Schrader and Merritt, 2000; Grueber et al., 2002, 2007) evidence suggests we will likely observe convergence among proprioceptor output synapses. However, we do not know to what extent output synapses will intermingle in space, nor what other features may organize their inputs. Therefore, we asked how proprioceptor synapses are organized, using the larval connectome to answer this question.

**FIGURE 1:**
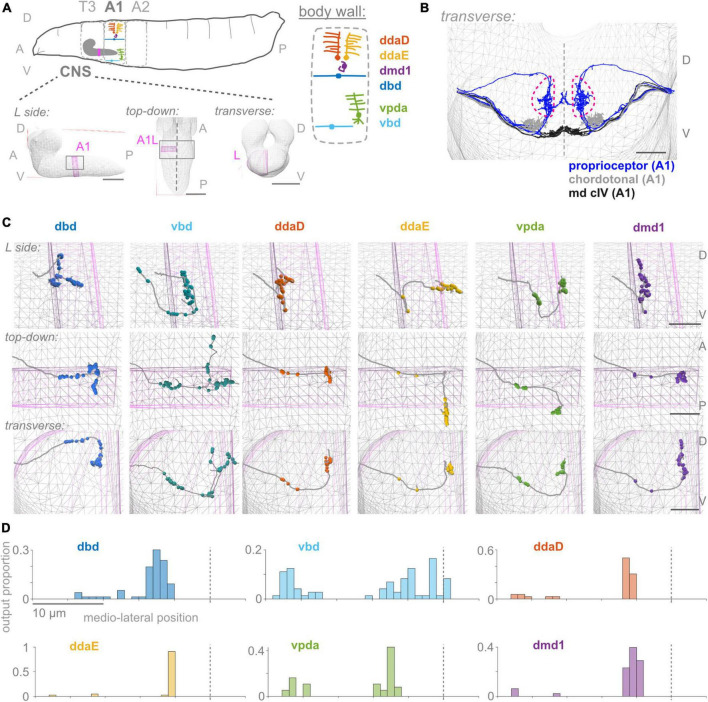
First abdominal segment proprioceptor output synapses are distributed along the proximal and distal axon. **(A)** Diagrams of larval body plan, of proprioceptive neuron somata and dendrites at the larval body wall (adapted from Cheng et al., 2010; Vaadia et al., 2019), and of views of the larval CNS. The same six proprioceptive neurons are present in each abdominal hemisegment (here, A1 left). They project to neuropil segment A1 in the CNS, highlighted in pink. **(B)** Transverse view of reconstructed skeletons (axonal projections) in A1 of the larval connectome: proprioceptive neurons (blue), chordotonal neurons (gray), and class IV multidendritic neurons (black). Dashed pink lines demarcate the “central domain” of proprioceptive outputs. Dashed gray line: midline. **(C)** Three views of output synapses in the CNS made by each left side proprioceptor. Gray lines: neuron skeletons. Spheres: output synapse locations. Top subpanels: left side views; middle subpanels: top-down views; bottom-subpanels: transverse views, looking from the tail toward the head. Gray mesh: outline of larval CNS. Pink mesh: outline of A1 left side region of neuropil. **(D)** Distribution of output synapses along the mediolateral axis for left side A1 neurons shown in **(C)**. Proportion of that neuron’s outputs made in 1 μm bins along the *X*-axis of the connectome. Views in **(B,C)** generated using CATMAID (Saalfeld et al., 2009). In **(A)**: scale bars = 50 μm; **(B,C)**: scale bars = 10 μm; scale bars equal for all axes. Throughout, dashed vertical lines: midline. D, dorsal; V, ventral; A, anterior; P, posterior; L, left side; T3, A1, A2, body segment abbreviations.

**FIGURE 2:**
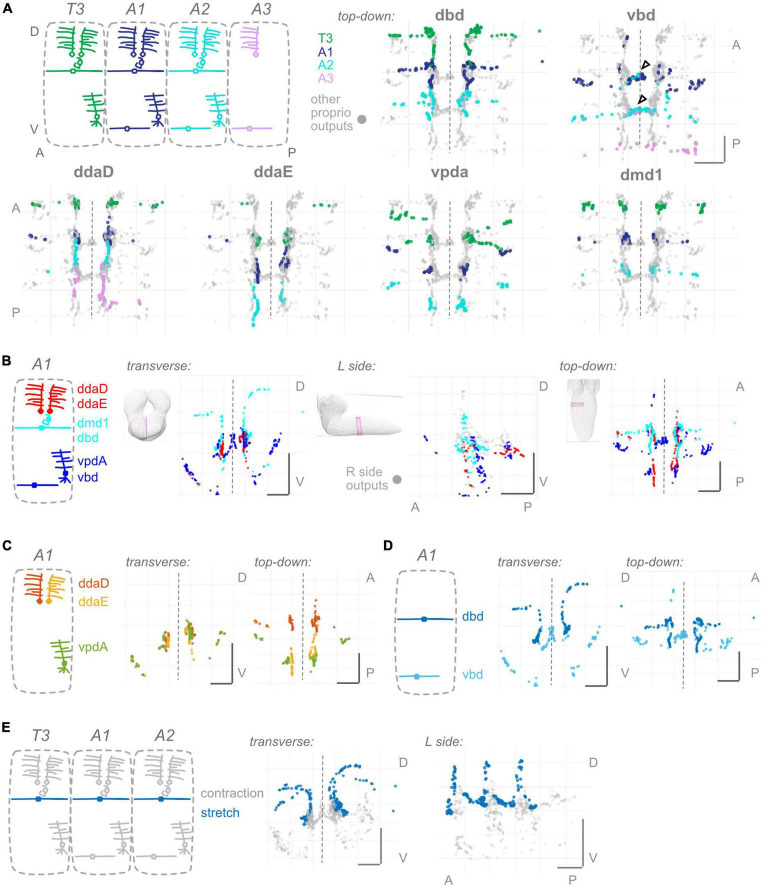
Proprioceptors show hemisegmental, but not dorsoventral or within-class, somatotopy. **(A)** Each proprioceptive neuron’s output synapses from segments T3–A2 or T3–A3 (for ddaD and vbd). Diagram: color scheme for neurons’ outputs from each segment; all other proprioceptor outputs plotted in gray. Note that vbd does not exist in T3. Top-down views are shown for each neuron type individually. Both left and right side neurons’ outputs are plotted. Black (unfilled) arrowheads show locations of segmental overlap for vbd neurons. Scale bars are same for all views. **(B)** Three views of output synapses from A1 proprioceptive neurons colored by approximate dorsoventral dendrite position at the body wall (diagram). For left side view only, right side neurons are plotted in gray. **(C)** Two views of output synapses from A1 class I multidendritic neurons, colored according to diagram. **(D)** Two views of output synapses from A1 bipolar dendrite class neurons, colored according to diagram. **(E)** Two views of output synapses from stretch-sensing T3–A2 dbd neurons (blue), compared to contraction-sensing proprioceptor output synapses (gray). Throughout: scale bars = 10 μm. Dashed vertical lines: midline.

**FIGURE 3:**
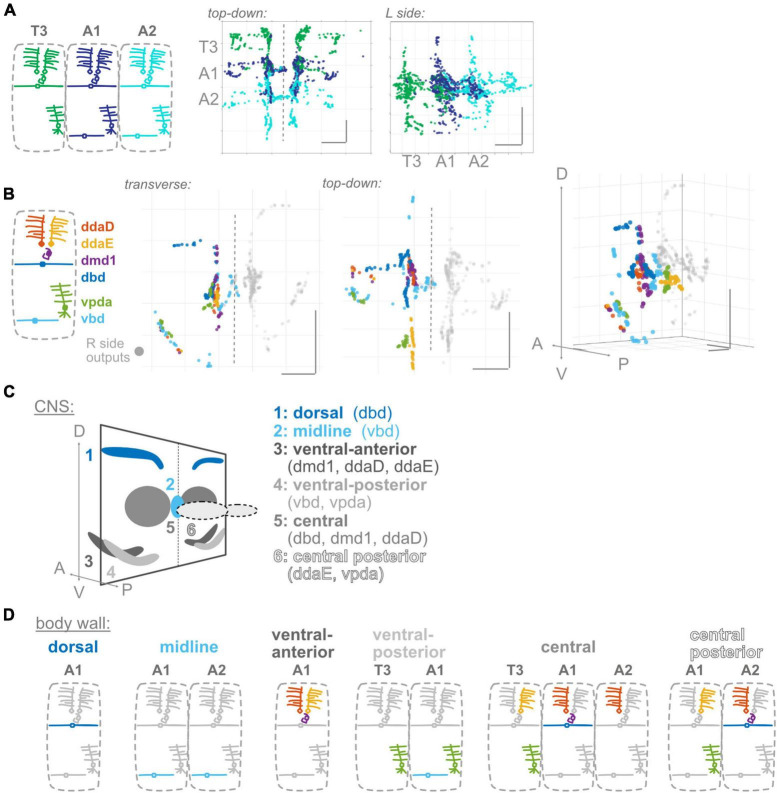
Proprioceptor outputs converge in unique combinations in multiple spatial regions. **(A)** Two views of overlap in output synapses from segments T3–A2, colored according to diagram. **(B)** Three views of overlap in output synapses from all six left side proprioceptors in A1, colored according to diagram. Right side outputs plotted in gray. **(C)** Summary diagram of six spatial domains where unique combinations of A1 proprioceptor outputs converge, names and contributing A1 proprioceptors given beneath image. Note that some of these domains will also contain outputs from T3 or A2 proprioceptors. **(D)** Corresponding dendritic “receptive fields” of the six spatial domains in **(C)**. Depicted for each spatial domain are the segment and cell type identity of the neurons that typically contribute output synapses in that area. Throughout: scale bars = 10 μm.

**FIGURE 4:**
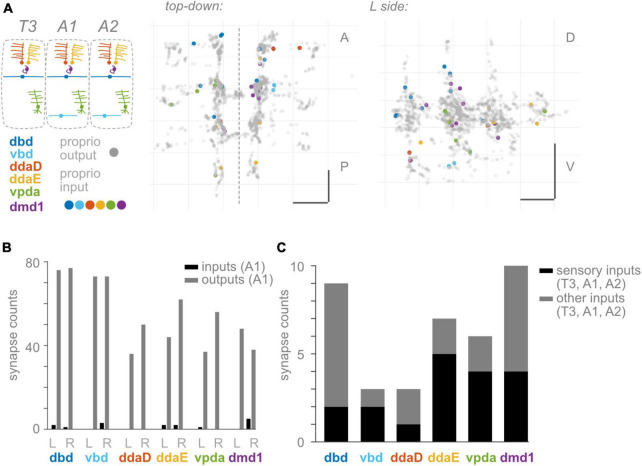
Proprioceptors receive few input synapses. **(A)** Two views of input synapses onto proprioceptive neurons in T3, A1, and A2, colored by cell type according to diagram. Output synapses from same neurons shown in gray. Scale bars = 10 μm. Dashed vertical line: midline. **(B)** Paired bar plots of input and output synapse counts for each proprioceptive neuron in A1. Dark bars: input synapse counts; light bars: output synapse counts. Counts are shown independently for left and right side A1 neuron of each type. **(C)** Stacked bar plot of input synapses onto each proprioceptive neuron, summed across T3, A1, and A2. Bottom bars (dark): total input synapses in T3-A2 from sensory neurons. Top bars (light): total input synapses in T3-A2 from other (non-sensory or unidentified) neurons.

**FIGURE 5:**
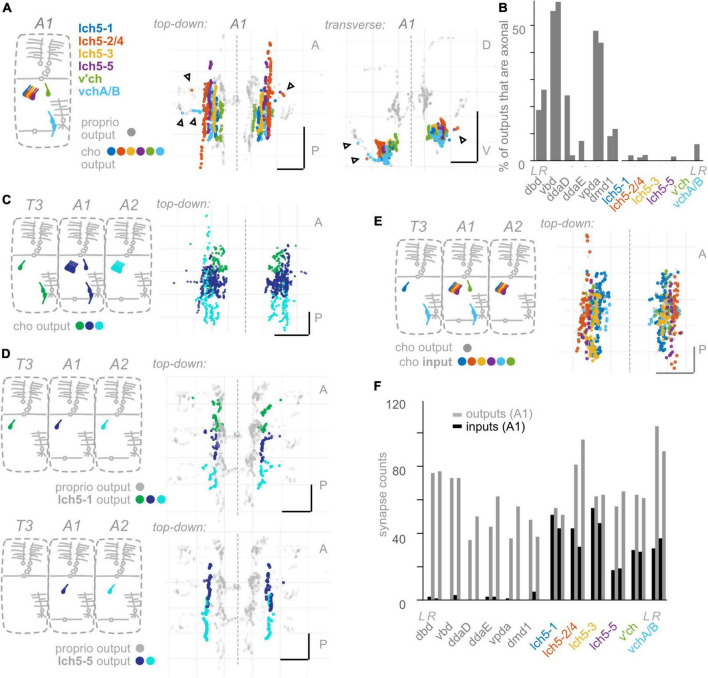
Chordotonal (cho) neurons make fewer axonal outputs than proprioceptors, show partial hemisegmental somatotopy, and receive more inputs than proprioceptors. **(A)** Diagram of cho dendrites at the body wall in segment A1, and two views of output synapses made by A1 cho neurons in the CNS, colored according to diagram. Black (unfilled) arrowheads indicate locations of axonal output synapses. **(B)** Percentage of all output synapses that are made along the proximal axon (see section “Materials and methods”) by each proprioceptive and cho neuron in A1. Left bars in each pair: left-side A1 neuron; right bars in each pair: right-side A1 neuron. **(C)** Top-down view of output synapses made by all cho neurons that have been reconstructed in segments T3, A1, and A2, colored according to diagram. **(D)** Top-down views of output synapse locations for two example cho neurons in segments T3, A1, and A2. Proprioceptor output synapses plotted in gray for comparison. **(E)** Top-down view of input synapses onto all cho neurons in segment A1, colored according to diagram. Output synapses made by the same neurons plotted in gray. **(F)** Paired, grouped bar plot of input and output synapse counts for each proprioceptive and chordotonal neuron in A1. Left bars in each pair (dark): input synapse counts; right bars in each pair (light): output synapse counts. Left pair of bars in each group: left-side A1 neuron; right pair of bars in each group: right-side A1 neuron. Throughout: scale bars = 10 μm. Dashed vertical lines: midline.

**FIGURE 6:**
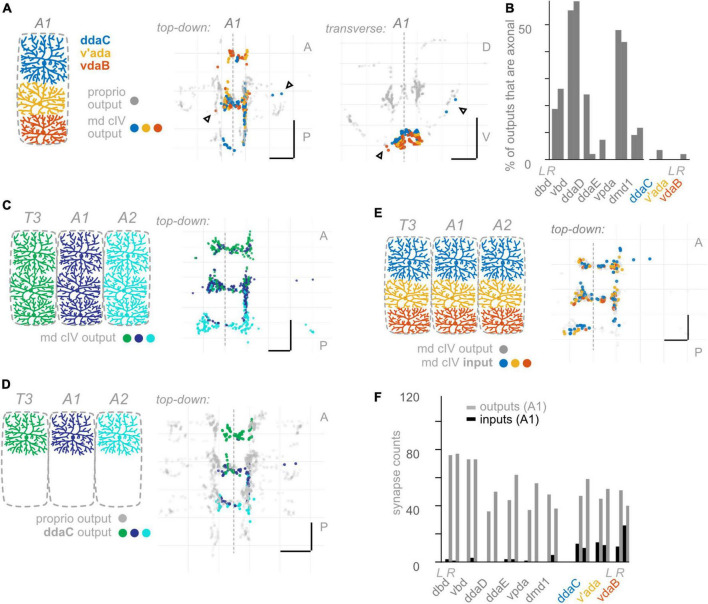
Class IV multidendritic (md cIV) neurons make fewer axonal outputs than proprioceptors, show no hemisegmental somatotopy, and receive more inputs than proprioceptors. **(A)** Diagram of md cIV dendrites at the body wall in segment A1, and two views of output synapses made by A1 md cIV neurons in the CNS, colored according to diagram. Black (unfilled) arrowheads indicate locations of axonal output synapses. **(B)** Percentage of all output synapses that are made along the proximal axon by each proprioceptive and md cIV neuron in A1. Left bars in each pair: left-side A1 neuron; right bars in each pair: right-side A1 neuron. **(C)** Top-down view of output synapses made by all md cIV neurons in segments T3, A1, and A2, colored according to diagram. **(D)** Top-down views of output synapse locations for the example md cIV neuron ddaC in segments T3, A1, and A2. Proprioceptor output synapses plotted in gray for comparison. **(E)** Top-down view of input synapses onto all md cIV neurons in segment A1, colored according to diagram. Output synapses made by the same neurons plotted in gray. **(F)** Paired bar plot of input and output synapse counts for each proprioceptive and md cIV neuron in A1. Left bars in each pair (dark): input synapse counts; right bars in each pair (light): output synapse counts. Left pair of bars in each group: left-side A1 neuron; right pair of bars in each group: right-side A1 neuron. Throughout: scale bars = 10 μm. Dashed vertical lines: midline.

In this study, we reviewed and added to the available connectomic data for larval proprioceptors (Ohyama et al., 2015). We focus on six proprioceptive neurons: dorsal bipolar dendrite (dbd), ventral bipolar dendrite (vbd), dorsal dendritic arbor D (ddaD), dorsal dendritic arbor E (ddaE), ventral posterior dendritic arbor (vpda), and dmd1 (Ghysen et al., 1986; Grueber et al., 2003). First, we describe output synapse locations; we find that, as a rule, *Drosophila* larval proprioceptors have synapses distributed along the incoming afferent, at both proximal and distal locations of the axon (Figure 1). We then map the distribution of proprioceptors in three consecutive body segments. Generally, the output synapses for each type of proprioceptor are organized according to a principle of left-right segmental somatotopy (Figure 2). Next, we map the overlap among output synapses from all proprioceptors within a single segment. *Drosophila* larval proprioceptor output synapses intermingle extensively in space; different combinations of output synapses contribute to six different spatial domains. Every proprioceptor type contributes synapses to at least two domains, and all but two domains have synapses from two or more proprioceptor types. This details the specific topography of convergence and divergence in the larval proprioceptive system (Figure 3). We also map proprioceptors’ input synapses, finding little evidence of presynaptic inhibition onto *Drosophila* larval proprioceptors (Figure 4). Finally, we compare the inputs and outputs of the proprioceptive neurons to other larval somatosensory neurons: those of chordotonal and class IV multidendritic neurons (Figures 5, 6). The principles of proximal axonal output, segmental somatotopy, and minimal presynaptic input do not apply broadly to larval somatosensory neurons. Thus, we have identified a suite of anatomical features that distinguish *Drosophila* larval proprioceptors from other larval somatosensors.

Taken together, our results reveal anatomical features that are potentially unique to larval proprioceptive processing. They open avenues to study the development and functional organization of proprioceptive circuits and offer an example of the utility of the larval connectome for uncovering fundamental cell biological observations.

## Materials and methods

### Dataset

The electron micrograph/connectome dataset used in this study is a CNS reconstruction from a 6-h-old first-instar larva, first described in Ohyama et al. (2015).

### Defining proprioceptive, chordotonal, class IV multidendritic, motor neurons

In general, we used the names assigned by annotators to assign reconstructed skeletons and the nodes attached to them into classes, as follows: *Proprioceptive neurons:* names beginning with “dbd,” “vbd,” “ddaD,” “ddaE,” “vpda,” or “dmd1.” *Chordotonal neurons:* names beginning with “lch5,” “v’ch,” or “vch.” *md cIV neurons:* names beginning with “ddaC,” “v’ada,” or “vdaB.” *Motor neurons:* names beginning with “MN,” which we further restricted to completely reconstructed, published motor neurons from segment A1 (see below).

We report on 138 total neurons. 104 were previously published, and 34 are new to this publication. See Supplementary Table 1 for details. To confirm the identities of unpublished neurons, we compared their morphology to light level examples (Merritt and Whitington, 1995; Schrader and Merritt, 2000; Grueber et al., 2002, 2007). For details of matching neurons in light-level data, see both Wreden et al. (2017) and Wang et al. (2022). In addition, we compared each neuron in other segments to the A1 example of the same name and to its left-right hemisegment sister neuron. The skeletons of identified neurons were reviewed to greater than 90% (with the exception of some published motor neurons).

### Data processing and analyses

Connectomic information, including synapse (”node”) locations and presynaptic and postsynaptic neuron identities, was extracted from CATMAID using the pymaid library.^1^

Visual representations of synapse locations were made either using MATLAB or using CATMAID’s 3D viewer tool. All “output” node locations plotted were presynaptic node locations (corresponding to the location annotated for the sensory neuron). All “input” node locations plotted, conversely, were postsynaptic node locations.

Other analyses were performed using MATLAB. MATLAB code for the visualizations and analyses will be made available on our Github.^2^

### Defining segment and side

We restricted visualizations and analyses using information about a neuron’s segment and side of origin, which we likewise obtained from the names assigned by annotators. We searched names for a tag containing segment and side, e.g., “_a1r.” For all counts of input and output synapses, neurons were restricted either to those in T3, A1, and A2, or to those in A1 alone.

### Defining sensory inputs

To obtain the population of sensory neurons that synapse onto proprioceptors/chordotonals/md cIVs (Figures 4–6), we defined sensory neurons as follows:

1. Any proprioceptor, chordotonal, or md cIV neuron, as defined above.
2. Additionally include any “es” neuron: any names beginning with “les,” “v’es,” or “ves.”
3. Additionally include any miscellaneous (partially identified) sensory neuron: any names containing the terms “(class I),” “(class 1),” or “sensory.”

Finally, we manually inspected the list of returned neuron names that met these criteria, to ensure that no obviously non-sensory neurons were accidentally returned.

### Defining spatial domains

Spatial domains formed by A1 output synapses were determined by eye, using the following criteria:

*Dorsal domain:* all synapses proximal to where the dbd axon projects ventrally, in a more-or-less vertical line (See below for corresponding coordinates).

*Ventral domains:* all synapses proximal to where the axons turns to project dorsally (or anteriorly, in the case of the vbd axon) (See below for corresponding coordinates). A gap of about one micron in the Z (A-P) axis separates the clusters of synapses assigned to ventral-anterior vs. ventral-posterior domains.

On the left side, A1L synapses assigned to any of the dorsal or ventral axonal domains correspond to those lateral to (greater than) *X* = 65000 nm, in CATMAID coordinates.

On the right side, A1R synapses assigned to the dorsal domain correspond to those dorsal to (less than) *Y* = 67000 nm. A1R synapses assigned to the two ventral domains lie lateral and ventral to a line drawn through (48000, 85000) and (38000, 75000) in the X, Y plane.

*Midline domain:* all synapses medial to the major antero-posterior tracts of the central domains. This corresponds to synapses with X positions between 53000 and 58500.

*Central domain:* synapses distal to the dorsal or ventral domains that group together in a cluster toward the medial part of the A1 neuropil.

This corresponds to synapses encompassed by the following ranges: X positions between 58500 and 65000 (L), or between 47000 and 53000 (R); Y positions (D-V) between 68000 and 84000; Z positions between 115000 and 125000.

*Central posterior domain:* all synapses posterior to the A1 segment of neuropil, with Z positions posterior to (greater than) 130000.

## Results

### Output synapses of *Drosophila* larval proprioceptors show a complex spatial organization

Proprioceptors provide essential feedback about the body’s movements, but how this information is incorporated into central proprioceptive processing circuits remains poorly understood. Here, we ask what principles underlie the spatial organization of proprioceptor input and output synapses in *Drosophila* larvae. To begin, we review specifics of the *Drosophila* larval body and how it moves: *Drosophila* larvae are left-right symmetrical and segmented, with much of the muscular and sensory anatomy repeated across body segments (Figure 1A; Ghysen et al., 1986; Bodmer and Jan, 1987; Campos-Ortega and Hartenstein, 1997). All three major modes of locomotion in *Drosophila* larvae—forward crawling, reverse crawling, and lateral rolling—involve sequential contraction of adjacent segments (Heckscher et al., 2012; He et al., 2022; Cooney et al., 2023). Segments are grouped into regions, including the anterior thorax with segments T1–T3 and the midbody abdomen with segments A1-A7. The nerve cord mirrors this organization: neuronal somata and axons and dendrite-rich central neuropil are grouped into regions corresponding to each body segment.

**FIGURE 7:**
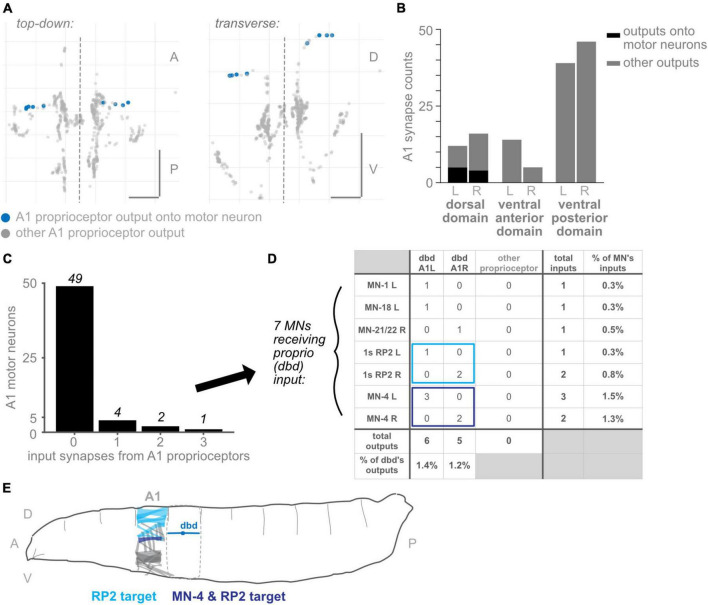
Subset of dorsal domain output synapses form monosynaptic reflex arcs onto motor neurons. **(A)** Two views of all output synapses made by A1 proprioceptive neurons. Synapses onto motor neurons colored in blue. **(B)** Stacked bar plot of output synapses in the three proximal axonal domains. Darker bars: synapses that contact motor neurons. Lighter bars: synapses that do not contact motor neurons. Counts for left- and right-side domains given separately. **(C)** Counts of A1 motor neurons receiving 0, 1, 2, or 3 input synapses from proprioceptors. No motor neuron received more than 3 such input synapses. **(D)** Table of proprioceptor synapse counts onto the seven motor neurons receiving direct proprioceptor input. Note that dbd accounts for all proprioceptor input. Rightmost column: percentage of all input synapses onto the motor neurons that come from dbdL/R. Bottom row: percentage of all output synapses made by dbd L and dbd R that are onto motor neurons. **(E)** Diagram of muscle targets of the two A1 motor neurons receiving bilateral proprioceptor input. Lighter blue: RP2 targets, dorsal muscles 1, 2, 3, 4, 9, 10, 19, 20 (Hoang and Chiba, 2001). Darker blue: MN-4 target, dorsal muscle 4. Although dbd is shown for reference in the adjacent segment, dbd’s motor neuron targets (and their muscle targets) are all located in the same segment. Throughout: scale bars = 10 μm. Dashed vertical lines: midline.

To monitor the movement of the larval body, in the periphery, each body segment contains left-right pairs of somata and dendrites belonging to six proprioceptors: ddaD, ddaE, vpda, dbd, vbd, and dmd1. The neurons from each segment initially project into that segment’s region of the neuropil (Figure 1A). Within the neuropil, proprioceptive neurons terminate in a region more dorsal compared to other somatosensory neurons’ projections, herein the **”central domain”** (Figure 1B; Merritt and Whitington, 1995; Schrader and Merritt, 2000; Landgraf et al., 2003). The available *Drosophila* larval connectome (Ohyama et al., 2015) allows the discovery of proprioceptor synapse locations with nanometer resolution. In the connectome, proprioceptor axons and synapses in A1 were already reconstructed (Heckscher et al., 2015; Schneider-Mizell et al., 2016; Zarin et al., 2019b). Due to the intersegmental nature of *Drosophila* locomotion (Heckscher et al., 2012), however, we need a map of proprioceptor synapses from multiple adjacent segments. Here, we reviewed the existing A1 annotations and added T3, A2, and in some cases, A3 proprioceptive neurons (see Figure 2). We sought to understand the spatial organization of proprioceptor output synapses through the following main lenses: individual proprioceptors within a segment (Figure 1); comparing individual proprioceptor types across segments (Figure 2); what information converges in spatial domains of the neuropil (Figure 3); inputs onto proprioceptors (Figure 4); and which features are common to *Drosophila* larval somatosensory neurons and which are unique to proprioceptors (Figures 5, 6). Finally, we examined the synapses from proprioceptors onto motor neurons to understand whether features of proprioceptor output synapse organization are predicted by the locations of monosynaptic sensorimotor reflex arcs (Figure 7).

### Proprioceptor output synapses are distributed along the length of the axon

To begin our characterization, we describe the positions of output synapses made by each A1 proprioceptor (Figures 1C, D). We looked for the distribution of outputs along the axon and whether synapses respect approximate midline and segment boundaries. The six proprioceptors under study belong to three morphological classes, which we use to organize the following description of their outputs. We give counts for the left side neuron of each left-right pair, although right side counts are similar (Supplementary Table 2).

Two proprioceptor types belong to the bipolar dendrite class, dbd and vbd; these have dorsal and ventral dendrites, respectively. dbd is the only proprioceptor to project into the neuropil along a dorsal route (Figure 1; Schrader and Merritt, 2000), and is also the only stretch receptor of the six (Tamarkin and Levine, 1996; Simon and Trimmer, 2009; Suslak and Jarman, 2015; He et al., 2019; Vaadia et al., 2019). dbd projects medially along the dorsal route until it reaches the proprioceptor domain. It then descends ventrally and bifurcates, sending branches both anterior (into neuropil segment T3) and posterior (Schrader and Merritt, 2007). dbd A1L makes 12 of its synapses (16%) along the proximal axonal entry route, with the remaining synapses in the dorsal portion of the A1 central domain. vbd projects into the neuropil along the more posterior of two ventral entry routes. While still ventral to the central domain, vbd bifurcates, sending one branch anterior (into neuropil segment T3), and the other dorsal toward the midline of A1, which it crosses. vbd is the only proprioceptor to cross the midline. vbd A1L makes 27 of its synapses (37%) along the proximal axonal entry route, 8 of its synapses ventrally at a medial location on the way to T3, 8 anteriorly in segment T3, 10 in the A1 proprioceptor domain, and the remaining 20 near the A1 midline. We conclude that neurons of the bipolar dendrite class distribute their synapses along both the proximal and distal axons upon entering the neuropil.

Three proprioceptor types belong to the class I multidendritic neurons (herein md cI), ddaD, ddaE, and vpda; these have dorsal-posterior, dorsal-anterior, and ventral dendrites, respectively (Figure 1A; Grueber et al., 2007). ddaD and ddaE project into the neuropil along the anterior of the two ventral entry routes. Both project medially until the central domain, then turn and project dorsally. At this point, ddaD makes synapses in the A1 central domain, while ddaE turns again and projects posteriorly, making synapses in the A2 central domain (Supplementary Figure 1). ddaD and ddaE A1L both make synapses along their proximal entry route (19 and 7% of their total output synapses, respectively), but tend to make fewer relative to the other proprioceptors (Figures 1C, 2A; see also Figure 5B). vpda projects into the neuropil along the posterior of the two ventral entry routes and projects medially until the A1 central domain. Then, it turns dorsally and again posteriorly, making synapses in the A2 central domain. vpda A1L makes 12 of its synapses (32%) along the proximal entry route and the remaining synapses posterior to the A1 central domain. We conclude that, like the bipolar dendrite class of neurons, md cI proprioceptors also have synapses along both proximal and distal axons.

One proprioceptor type, called dmd1, is not grouped into morphological classes with the other neurons (Grueber et al., 2003). dmd1 projects into the neuropil along the anterior of the two ventral entry routes, then medially until the A1 central domain, and again dorsally. dmd1 A1L makes most of its synapses in a narrow dorsoventral column that stays within the A1 central domain, with the remaining 4 synapses (10%) along the proximal entry route. We conclude that dmd1 proprioceptors also have synapses along both proximal and distal axons.

#### Do output synapse locations differ in other segments?

In *Drosophila* larvae, anterior-posterior differences exist among body segment muscles and sensory neurons (Ghysen et al., 1986; Bodmer and Jan, 1987; Campos-Ortega and Hartenstein, 1997). Moreover, different body segments can participate differently in larval behaviors (Lahiri et al., 2011; Heckscher et al., 2012). Therefore, there are likely to be differences in proprioceptor output synapse number or organization between segments. We find the following differences: first, segment T3 lacks a vbd neuron. Second, dmd1 neurons from T3 make more synapses along the proximal axon entry route than abdominal dmd1 neurons. Third, vpda neurons from T3 make more lateral synapses than abdominal vpda neurons. Lastly, ddaD neurons from A2 follow a different pattern than those from A1 or T3, projecting anteriorly and making synapses extending into the central domain of A1. ddaD from A3 shows a pattern similar to ddaD from A2. In contrast, three proprioceptors—dbd, ddaE, and dmd1—did not display segment-specific characteristics. We conclude that a subset of proprioceptors has segmentally specific output synapses, and this segmental specificity is distinctive for each neuron.

In summary, proprioceptor output synapses are distributed along the length of the axon. This distribution is most often into two groups: typically, a smaller set of output synapses along the proximal axon and a higher concentration of output synapses at the distal tips. Two of the six (ddaD and dmd1) respect A1 hemisegmental bounds, while the other four extend beyond the anterior or posterior boundary of A1, and vbd alone crosses the midline (Figure 1C). Additionally, the output synapse distributions seen in A1 are generally representative, but certain proprioceptors display segment-specific features (Figure 2A).

### Proprioceptor output synapses, regardless of type, morphological class, or function, show hemisegmental somatotopy

Having characterized output synapse locations for individual proprioceptors from a single segment and compared these across segments, we next compared within-segment output synapse locations across proprioceptor types. We looked for spatial organization in any of the following forms: (1) Many sensory systems display a form of somatotopy in which relative sensor positions are maintained centrally. Since different larval proprioceptors monitor different dorsal-ventral positions within a segment’s body wall, we might see a corresponding map of output synapses in the neuropil. (2) Beyond somatotopy within a segment, we might see somatotopic organization across segments, since segments can be considered anterior-posterior “units” of the larval body. We might additionally see organization that keeps the information from each side of the body (left and right) spatially separated, potentially to be used in left-right symmetrical circuits. (3) Outputs may be organized within a morphological class (e.g., bipolar dendrite neurons). In the peripheral body wall, somatosensory dendrites from the same morphological class send dendrites to different regions of space by a mechanism of active avoidance (Grueber and Sagasti, 2010). Analogous mechanisms may separate within-class outputs in the CNS. (4) Outputs may be organized by function. In the case of larval proprioceptive neurons, function and morphological class are not synonymous (He et al., 2019; Vaadia et al., 2019). Here, we ask which of the above principles, if any, govern where individual proprioceptors form their output synapses.

#### 1: no evidence for dorsal-ventral somatotopy within segments

Because the larval body wall is roughly two-dimensional, we consider somatosensory dendrites as having a Dorsal-Ventral (D-V) or Anterior-Posterior (A-P) mapping. Two of the proprioceptors cover the full A-P extent of the segment with their dendrites; these could not readily be classified as “anterior” or “posterior.” Therefore, we compare all proprioceptors within a segment, focusing on D-V mapping. We grouped the six proprioceptors into three groups based on their relative dendrite positions. All groups intermingle extensively in the A1 central domain (Figure 2B and Supplementary Figure 2). We did not observe any axis along which there was a conservation of order from the periphery. We conclude that there is no evidence of a dorsoventral somatotopy among proprioceptors.

#### 2: proprioceptor synapses display hemisegmental somatotopy

Here, we looked for somatotopy at the level of hemisegments, where a hemisegment refers to the right or left half of a single segment. Comparing the output synapse locations of T3, A1, and A2 segment proprioceptors, all individual dorsal proprioceptor types (dbd, ddaD, ddaE, and dmd1) display little overlap (Figure 2A and Supplementary Figure 3). Ventral proprioceptors are an exception: (1) vbd synapses from A1 and A2 overlap in the A1 midline (Figure 2A, arrowheads), and this pattern continues for vbd synapses from A2 and A3. (2) vpda synapses from T3 and A1 overlap in a ventral-lateral region (Figure 2A), but the pattern does not continue further posterior. We conclude that, in general, individual proprioceptors of the same type have little overlap in their output synapse positions and thus observe hemisegmental somatotopy.

#### 3: bipolar dendrite but not multi-dendritic class I output synapses are separated within their morphological class

Proprioceptors comprise multiple morphological classes. At the body wall, dendrites from neurons within a morphological class are well separated. Here, we ask whether a similar separation applies to the outputs within the CNS. Among the morphological class of md cI neurons (ddaE, ddaD, vpda), output synapses intermingle extensively (Figure 2C). ddaD and ddaE synapses overlap in the ventral region of their shared axonal entry route. Within the central domain, ddaD synapses also overlap extensively with ddaE and vpda synapses from the next anterior segment (Supplementary Figure 1). By contrast, among bipolar dendrite neurons, outputs are largely separated: dbd outputs remain at the dorsal margins of the central domain, while most vbd outputs are either more ventral or medial than dbd outputs (Figure 2D). Overall, we conclude that within-class separation of output synapse locations is seen for bipolar dendrite, but not for md cI proprioceptors, and thus does not represent a general principle in organizing proprioceptor outputs.

#### 4: stretch receptors partially separate their outputs from those of contraction receptors

Proprioceptive synapses might be organized centrally following their function, rather than by morphological class *per se*. In many systems, morphological class and function are linked (Tuthill and Azim, 2018), but in larval *Drosophila*, they are not. For instance, dbd and vbd are in the same morphological class, but dbd is the only known stretch receptor among all proprioceptors, including vbd (Vaadia et al., 2019). We compared dbd’s output synapse locations to those of the remaining five, contraction-sensing proprioceptors. dbd’s outputs along the dorsal axon entry route are well separated from those of other proprioceptors (Figure 2E). By contrast, the remainder of dbd’s outputs are in the A1 central domain, in close proximity to outputs of ddaD, dmd1, and occasionally vbd. Therefore, we see a partial separation of stretch and contraction neuron outputs.

Overall, the main organizing principle that applies to all proprioceptors, regardless of type, morphological class, or function, is hemisegmental somatotopy.

### Proprioceptor output synapses display a convergent and divergent spatial organization

Information from a single proprioceptive sensor is rarely used in isolation; rather, combinations of proprioceptors are thought to convey information about the state of a body region (Bosco and Poppele, 2001; Jankowska, 2008). There is functional evidence in *Drosophila* larvae that proprioceptive information is used in combination (Hughes and Thomas, 2007). Further, given our observations (Figure 1) that proprioceptors distribute their synapses widely along their axons, including areas beyond the previously described “central domain” (Figure 1; Merritt and Whitington, 1995; Zlatic et al., 2009), we expect proprioceptive information is likely distributed and combined in space. We first examined adjacent segments to determine where synapse outputs overlap in space (Figure 3A). There is extensive interdigitation across segments, much of which seems to occur in the central domain region of each segment; e.g., outputs from both adjacent segments contribute to the A1 central domain. We next looked within a single hemisegment (A1 L or R) at the outputs of the six proprioceptors from that hemisegment to identify where these outputs converge (Figure 3B). We noticed convergence in the A1 central domain and in multiple locations more ventrolateral and more posterior. Finally, we combined these approaches to determine which neurons contribute to the cross-segment overlap and in which spatial domains (Figure 3C), described below.

A1 proprioceptors contribute to six spatial domains (some of which also contain outputs from T3 and/or A2 proprioceptors). For each domain, based on the neurons that contribute output synapses, we reconstructed the domain’s expected “receptive field” at the body wall (Figure 3D). The domains, their contributing proprioceptors, and predicted receptive fields are as follows: (1) The **dorsal domain** in A1 receives information from only one A1 proprioceptor (dbd), and forms along dbd’s dorsal axonal entry route; this domain is predicted to contain information about segment stretch. (2) The **midline domain** in A1 also receives information from only one A1 proprioceptor (vbd), but forms at the distal tips of vbd axons rather than along the proximal entry route. A2 vbd also contributes to this domain. The midline domain is predicted to contain bilateral information about ventral contraction. In contrast to the dorsal and midline domains, (3) the **ventral-anterior** and (4) **ventral-posterior** domains in A1 receive information from multiple proprioceptors. These domains, which are along the ventral axonal entry routes, are predicted to contain information about dorsal contraction and ventral contraction, respectively. (5) The **central domain** receives information from multiple A1 proprioceptors and proprioceptors originating in adjacent body segments. This combination of outputs is predicted to contain a complex mixture of information about the contraction of multiple segment boundaries; because two of the contributing neurons are direction-selective (He et al., 2019; Vaadia et al., 2019), the activity of outputs in this domain likely depends on the direction of movement. (6) The **central posterior domain** is the final domain to contain synapses from A1 proprioceptors; it is the equivalent of the A1 central domain but in the next posterior segment (A2). The same predictions hold for this domain’s receptive field and the information it contains.

In general, we find complex patterns of proprioceptive information converging in space, often along the axonal entry routes. Each proprioceptor contributes outputs to multiple spatial domains, and most spatial domains contain only proprioceptive information from within the segment, but the central domain contains information from multiple adjacent segments.

### Few presynaptic inputs onto *Drosophila* larval proprioceptors

Presynaptic inhibition onto proprioceptive afferents is widespread across species (Clarac and Cattaert, 1996; Rudomin and Schmidt, 1999; Azim and Seki, 2019). In many cases, proprioceptive circuits use inhibition to scale the magnitude of incoming sensory signals, which may be needed to encode a wide dynamic range and/or avoid fatiguing the sensors (Straka et al., 2018; Tuthill and Azim, 2018; Skandalis et al., 2021; Gebehart et al., 2022). In addition, inhibition of proprioceptive feedback may be needed to modify motor reflexes such that they do not occur at inappropriate times (Eccles et al., 1962; Burrows, 1996; Rossignol et al., 2006; Fink et al., 2014). Therefore, presynaptic inhibition is widely thought to be necessary for proprioception, and it is reasonable to expect presynaptic inhibition of *Drosophila* proprioceptors. However, remarkably, proprioceptors in A1 reportedly receive few presynaptic inputs (Schneider-Mizell et al., 2016). To confirm and extend these findings, we looked for presynaptic inputs onto *Drosophila* larval proprioceptors in other segments. Where possible, we asked if inputs were likely to be excitatory or inhibitory. Compared to inputs, we confirm A1 proprioceptors receive few inputs (Figures 4A, B). In A1, the most input synapses received by any single proprioceptor is five (onto R side dmd1; Figure 4B). This pattern holds true across segments (Supplementary Table 2). Moreover, multiple proprioceptors do not appear to receive any input synapses. On average, a proprioceptor’s number of inputs is only 2% of the number of its outputs. Indeed, the 34 proprioceptive neurons in segments T3, A1, and A2 receive only 38 synaptic inputs between them (Figure 4A). We find that the 38 synaptic inputs come from 32 presynaptic neurons, at least 15 of which are other sensory neurons (Figure 4C). *Drosophila* somatosensory neurons are expected to be excitatory (Ohyama et al., 2015). In summary, we confirm there are few input synapses onto proprioceptive neurons. Additionally, we find that many presynaptic partners are most likely excitatory. Overall, there is little evidence for pre-synaptic inhibition of larval proprioceptors.

### Comparisons among somatosensory modalities

So far, we have uncovered three features that describe the anatomy of *Drosophila* larval proprioceptors: (1) they form output synapses along axon routes into the nerve cord; (2) they receive few presynaptic inputs; and (3) individual proprioceptor types follow the principle of hemisegmental somatotopy. Given that features 1 and 2 are both surprising and potentially functionally significant (Clarac and Cattaert, 1996; Cover and Mathur, 2020), we next wanted to see whether these features were common to other *Drosophila* larval somatosensors. Therefore, we looked for the above three features in two additional classes of *Drosophila* larval somatosensory neurons (Figure 1B): chordotonal neurons (“chordotonals”) and class IV multidendritic neurons (”md cIVs”) (Ghysen et al., 1986; Bodmer and Jan, 1987; Grueber et al., 2002), which have been annotated in the connectome by other groups (Ohyama et al., 2015; Jovanic et al., 2016). The chordotonals in abdominal body segments comprise seven neurons: five lateral chordotonal (lch) neurons, found in a cluster together at the body wall (Figure 5A); one additional lateral chordotonal (v’ch); and two ventral chordotonals (vch) (Ghysen et al., 1986). The md cIV neurons in abdominal body segments comprise three neurons; their highly elaborate dendritic arbors tile most of the body surface (Figure 6A). Previous work has characterized their axonal projections into the neuropil (Merritt and Whitington, 1995; Schrader and Merritt, 2000; Grueber et al., 2007; Zlatic et al., 2009). Here, we evaluate the spatial organization of their output synapses, focusing on comparing patterns to those found in proprioceptors.

#### Compared to proprioceptors, other somatosensory neurons make fewer output synapses along the proximal-distal axis of their central axon

Having observed that proprioceptors form a substantial fraction of their output synapses along axonal entry routes, we looked for the presence of chordotonal and md cIV output synapses along these neurons’ axonal entry routes. From the chordotonal neurons in A1, we counted 11 output synapses along the incoming axon, compared to 846 total outputs (1.3%) (Figures 5A, B). This percentage remains at 1.0% when including chordotonals in segments T3–A2. Meanwhile, from the md cIV neurons in A1, we counted 4 output synapses along the incoming axon, compared to 294 total outputs (1.3%) (Figures 6A, B), and this percentage remains at 1.4% when including md cIV neurons in T3–A2. We conclude that, compared to proprioceptors, both chordotonals and md IV neurons make few output synapses along their axon routes into the central neuropil. The synapses along the axons thus appear to be a feature specific to proprioceptors.

#### Comparison of somatotopic organization of neurons from one class

We next asked whether hemisegmental somatotopy applies to chordotonal neurons. First, we characterized the outputs of left-right chordotonals originating in segment A1. Chordotonal synapses are organized into columns that run anteroposterior, closely resembling their previously characterized axonal trajectories (Merritt and Whitington, 1995). The outputs from A1 chordotonal neurons largely remain within the A1 neuropil segment and never cross the midline (Figure 5A). This columnar form of spatial organization is unlike any form of organization displayed by the *Drosophila* larval proprioceptors. Next, we mapped output synapses from chordotonal neurons in three adjacent segments, T3–A2. Some, but not all, chordotonal neurons in these segments restrict their outputs to their own segment of neuropil (Figures 5C, D and Supplementary Figure 4); We also found extensive overlap across segments for many chordotonals originating in segments posterior to A2 (not shown). However, hemisegmental somatotopy is present for a subset of chordotonal neurons (lch5-1 and lch5-3: Supplementary Figure 4). We conclude that hemisegmental somatotopy is less common among chordotonals compared to proprioceptors.

We performed the same set of analyses for md cIV neurons. First, outputs from all three md cIV neurons originating in segment A1 intermingle extensively (Figure 6A). This is reminiscent of the spatial organization of proprioceptors belonging to md cI (ddaD, ddaE, and vpda), but not other types of proprioceptors. Further, md cIV neurons rarely restrict their outputs to a single segment of neuropil (Figures 6C, D and Supplementary Figure 5); instead, outputs from adjacent segments overlap both lateral to and at/across the midline (Supplementary Figure 5). We checked this pattern for md cIV neurons annotated in other segments thus far and confirmed that the general rule is for a neuron to form synapses across its own segment of neuropil, plus at least one adjacent segment (not shown). We therefore see no evidence of hemisegmental somatotopy among md cIVs, in contrast to proprioceptors.

We conclude that hemisegmental somatotopy is present for outputs from a subset of chordotonal neurons and absent for outputs from md cIV neurons. This feature, which was common among proprioceptors (except for vbd), is therefore not a general feature of somatosensory neurons.

#### Most *Drosophila* larval somatosensory neurons have abundant presynaptic input

As noted earlier, presynaptic input onto sensory neurons is a common feature of many sensory systems. However, there are a small number of presynaptic inputs onto larval proprioceptors (Figure 4B). To test whether this small number of input synapses is a general feature of larval somatosensory processing, we investigated the inputs onto chordotonal and md cIV neurons. These analyses confirm and extend those of Jovanic et al. (2016) and Gerhard et al. (2017). First, we visualized the input synapses onto chordotonal and md cIV neurons in segments T3, A1, and A2. Compared to proprioceptors (Figure 4A), chordotonal and md cIV neurons receive input synapses broadly throughout their arbors (Figures 5E, 6E). Next, we quantified the total input and output synapses for chordotonal and md cIV neurons in segment A1, as this segment has a complete set of reconstructed sensory neurons (Figures 5F, 6F and Supplementary Table 3). While overall numbers of output synapses are comparable in magnitude across each of the three classes— proprioceptors, chordotonals and md cVIs—the numbers of input synapses are much smaller among proprioceptors than among the other two classes. As a result, the ratio of input to output synapses is also noticeably smaller for proprioceptors. While the mean input-to-output ratio for A1 proprioceptors (expressed as a percentage) is only 2.6%, for A1 chordotonals it is 54.4%, and for A1 md cIVs it is 30.9%. The relative lack of presynaptic input appears to be a unique feature of larval proprioceptors.

In summary, we identify unique anatomical features of larval proprioceptive neurons that set them apart from other somatosensors: (1) they form output synapses along axon routes into the nerve cord; (2) they receive very few presynaptic inputs; and (3) individual proprioceptor types follow the principle of hemisegmental somatotopy.

### A subset of dorsal proximal output synapses belong to monosynaptic reflex arcs from proprioceptors to motor neurons

Among the best-characterized circuits in neuroscience are monosynaptic reflex arcs between proprioceptors and motor neurons, which are important in many systems for the fast retraction or stabilization of limbs (Tuthill and Azim, 2018). However, in non-limbed animals, few direct connections between proprioceptors and motor neurons have been described. A notable exception is a caterpillar, *Manduca sexta*, in which proprioceptor-to-motor neuron direct connections have been reported (Tamarkin and Levine, 1996). Because the proximal output synapses are an unusual feature of proprioceptors (compared to chordotonals and md cIVs), we asked whether their presence might be accounted for by direct connections to motor neurons. In other words, are the three proximal domains (dorsal, ventral-anterior, and ventral-posterior domains) the locations of monosynaptic connections between sensory and motor neurons?

We looked for synapses from proprioceptors onto motor neurons from A1, the only segment in which motor neurons have been comprehensively reconstructed and annotated (Zarin et al., 2019b). Other authors have reported a small number of direct connections from dbd neurons to motor neurons (Schneider-Mizell et al., 2016; Zarin et al., 2019a); we reviewed these annotations to double-check for any missing proprioceptor to motor neuron synapses. We could find only one additional synapse from proprioceptors in A1 onto motor neurons, bringing the reported total from 10 to 11 (Figure 7). We confirmed that, in this EM dataset, no connections are reported between chordotonal or md IV neurons and motor neurons (data not shown).

We found that, of the three proximal domains, only synapses in the dorsal domain innervated A1 motor neurons (Figures 7A, B). As this domain exclusively contains output synapses from dbd, we confirmed that only dbd proprioceptive neurons directly synapse onto motor neurons in A1. Of the 12 proximal synapses in the left-side dorsal domain, 5 contact motor neurons, or 42%. (These 5 output synapses innervate 6 motor neurons; in *Drosophila* larvae, one pre-synapse typically contacts multiple post-synaptic neurons). Of the 16 proximal synapses in the right-side dorsal domain, 4 contact motor neurons, or 25%.

We conclude that synapses onto motor neurons can only account for a small fraction of the proprioceptors’ proximal axonal synapses, and only from dbd neurons.

#### Direct connections from proprioceptors to motor neurons show the expected connectivity for a monosynaptic reflex arc

We next asked whether the synapses from proprioceptors onto motor neurons show the expected connectivity for monosynaptic reflex arcs. Monosynaptic reflex arcs typically enable “corrective” reflexes: for instance, when proprioceptive feedback signaling an increase in muscle stretch (e.g., from muscle spindles) causes increased activation of motor neurons that innervate the muscle in question, counteracting the stretch (Rossignol et al., 2006). Similarly, since the only monosynaptic sensory-to-motor connections observed so far in larvae are from dbd neurons, which are dorsally positioned longitudinal stretch sensors (Schrader and Merritt, 2000; Suslak and Jarman, 2015), we might expect the motor neuron targets to be those that innervate dorsal longitudinal muscles, whose elongation would promote dbd activity and whose contraction would inhibit dbd activity.

We therefore evaluated which motor neurons received input from dbd in A1 R and L. Of all 56 motor neurons with complete annotations in A1, 49 received no dbd input (Figure 7C). A total of four motor neurons received a single input synapse, and only 3 received more than one input synapse. These synapse counts are low: dbd input accounted for no more than 1.5% of any motor neuron’s inputs, and conversely, synapses onto motor neurons (combined) accounted for less than 1.5% of all dbd output synapses.

The identities of the motor neurons receiving any dbd inputs are shown in Figure 7D. Only two motor neurons received dbd inputs on both sides of the midline: both left- and right-side RP2 and MN-4 motor neurons received synapses from the ipsilateral dbd neuron. Because they are consistent on both sides, we consider these synaptic partnerships more likely to be genetically pre-specified and to recur in other body segments (Ohyama et al., 2015). In support of this conjecture, we looked for the (incompletely reconstructed) RP2 motor neuron in two additional hemisegments, T3L and A2R, and confirmed the existence of a small number of synapses from dbd onto RP2 in these hemisegments (data not shown). Both MN-4 and RP2 show the expected connectivity for a stretch-countering reflex arc: MN-4 innervates a lateral longitudinal muscle close to dbd’s dendritic field. RP2, also known as the dorsal type Is motor neuron, innervates several dorsal muscles (Figure 7E). Activity of either motor neuron could, presumably, cause the body segment to shorten along the longitudinal axis and counter the stretch sensed by dbd.

Finally, we note that the three A1 motor neurons receiving dbd inputs on just one side of the midline, MN-1, MN-18, and MN-21/22, all innervate dorsal muscles (Hoang and Chiba, 2001); however, MN-18 and MN-21/22 innervate muscles with a transverse orientation, whose contraction would not directly counter longitudinal stretch.

We conclude that the synapses from dbds onto motor neurons in A1, while few in number, generally show the expected connectivity for a monosynaptic reflex arc.

## Discussion

Circuits that process proprioceptive information are essential to locomotor control. In this study, we describe the anatomical organization of the first stage of proprioceptive processing circuits: the input and output synapses of proprioceptors. We identified four anatomical features that differentiate *Drosophila* larval proprioceptors (Figures 1–4) from other somatosensory neurons (Figures 5, 6). (1) All *Drosophila* larval proprioceptors project to a region of the CNS that is dorsal to other somatosensory projections, in agreement with previous reports (Merritt and Whitington, 1995; Schrader and Merritt, 2000; Zlatic et al., 2009). All but vbd project to a common region, the “central domain” (Figures 1, 3). (2) Nearly all proprioceptor types display hemisegmental somatotopy, meaning their own outputs do not cross the midline and tend to repeat, but do not overlap, across adjacent segments (Figure 2). (3) *Drosophila* larval proprioceptors make proximal and distal output synapses along the axon, leading to the complex mapping of proprioceptive outputs into multiple spatial domains (Figures 1, 3). The presence of proximal output synapses in proprioceptors cannot be explained by these neurons’ connections to motor neurons (Figure 7). (4) *Drosophila* larval proprioceptors receive few presynaptic inputs, in agreement with previous reports (Schneider-Mizell et al., 2016), and we newly conclude that few inputs are inhibitory (Figure 4). In summary, we have described the spatial logic and distinctive features that characterize the organization of *Drosophila* larval proprioceptive synapses.

### Potential limitations of our mapping efforts

Here, we focus on six proprioceptive neurons: dbd, vbd, ddaD, ddaE, vpda, and dmd1 (Ghysen et al., 1986; Orgogozo and Grueber, 2005). We do not wish to argue that these are the only neurons that sense proprioceptive information in the *Drosophila* larva. We focus on this set because they are widely agreed to be proprioceptive in nature (Tamarkin and Levine, 1996; Simon and Trimmer, 2009; He et al., 2019; Vaadia et al., 2019). However, in adult insects, chordotonal neurons are proprioceptive (Burrows, 1996; Field and Matheson, 1998; Mamiya et al., 2018), and in larvae, chordotonal and md cIV neurons may respond to self-movement (Ainsley et al., 2003; Caldwell et al., 2003; Song et al., 2007). Significant anatomical differences exist between the six “proprioceptive neurons,” chordotonal neurons, and md cIV neurons. These differences do not rule out that chordotonal or md cIV neurons encode proprioceptive information, but they do raise questions about how different anatomies arise during development and how they contribute to different circuit-level properties.

This study takes advantage of an EM dataset of the larval CNS to gain nanometer-resolution insight into the cell biological, morphological, and spatial features of proprioceptive neurons. We use EM data to generate a detailed map of larval proprioceptor inputs and outputs across three body segments, the most complete map of its kind. However, EM datasets come with their own set of limitations (Morgan and Lichtman, 2013): first, we cannot assume synapses are functional or determine their strengths, which might lead us to overlook patterns related to functional connectivity. Second, reconstructions are subject to occasional annotation errors. Lastly, the EM dataset is from one animal at one developmental stage and may reflect idiosyncrasies in this animal’s development or transient patterns (e.g., synapses that will be pruned over time; although see Gerhard et al., 2017).

Finally, we have not described proprioceptors’ postsynaptic partners in this study, leaving open many important lines of follow-up inquiry (see below). The number of postsynaptic partners is likely to be in the hundreds, as in *Drosophila*, each presynaptic site can contact multiple postsynaptic sites, each potentially belonging to a unique neuron.

### Novel insights into proprioceptor output synaptic divergence and convergence

Proprioceptive processing circuits have long been thought to be characterized by the properties of divergence and convergence (Bosco and Poppele, 2001; Jankowska, 2008; Tuthill and Azim, 2018). At the anatomical level, divergence and convergence have largely been understood by examining the projection patterns of individual proprioceptor afferents, leading to identification of regions of the CNS innervated by different sensory types (Eccles et al., 1957; Murphey et al., 1985; Merritt and Whitington, 1995; Smith and Shepherd, 1996; Niu et al., 2013; Lai et al., 2016). In this study, thanks to the larval EM dataset, we can demonstrate the existence of both convergence and divergence at the level of proprioceptive outputs in space and describe patterns in how specific proprioceptor types converge and diverge at this level. These descriptions are a first step in unraveling the spatial complexity of the larval proprioceptive system and extracting organizational principles, discussed below.

#### Divergence

Anatomical divergence of larval proprioceptive output synapses is underpinned by two main strategies.

First, hemisegmental somatotopy segregates the outputs of (most) proprioceptive types across adjacent segments. Hemisegmental somatotopy may be important from the point of view of downstream partners. A limited number of downstream partners have been identified in other studies, including both local interneurons (e.g., Jaam neurons) and intersegmental interneurons (e.g., late-born Even-skipped Lateral interneurons; Heckscher et al., 2015). The presence of both local and intersegmental downstream partners implies that proprioceptive information is likely both to be used within a segment and to be simultaneously distributed to other segments. In the case of local downstream neurons, whose dendrites are largely restricted to one segment of neuropil, a matching restriction of partner proprioceptor synapses could help establish segmentally repeated circuits that are responsible for the local implementation of a proprioceptive processing computation (Thomas et al., 1984; Clark et al., 2018). Such local computations have been suggested for, e.g., computing a local bending angle or correcting left-right asymmetries (Heckscher et al., 2015; Vaadia et al., 2019). Other somatosensory neurons (e.g., nociceptors) do not respect the principle of hemisegmental somatotopy, implying that such local output restrictions may be more important for proprioceptive processing than for other somatosensory processing circuits.

Second, individual proprioceptors distribute output synapses along their axons at both proximal and distal locations. In the case of dbd, only the proximal synapses contact motor neurons (Figure 7), suggesting a potential separation of downstream partners by synapse location. However, we cannot currently determine whether this finding is the exception or the rule. Furthermore, most proprioceptor types distribute synapses into multiple domains, one of which is typically the central domain (Figure 3 for domain definitions). The exception is vbd, whose outputs are not distributed to the central domain but instead contribute to the midline domain. This raises the additional question of why vbd alone locates its outputs in this region: does it participate in distinct proprioceptive processing circuits from other proprioceptors? Future analysis of downstream partners should help resolve these questions.

#### Convergence

Anatomical convergence of output synapses reveals interesting patterns regarding what proprioceptive information could be combined in space. There are six proprioceptor types in *Drosophila* larvae, whose outputs could be spatially combined in many patterns. However, given the total number of possibilities, we find evidence for a rather limited number of combinations. Five domains consist of interdigitated outputs from multiple proprioceptor types (Figure 3). Only in the central and midline domains are outputs combined from multiple segments. Furthermore, in these domains, we do not see indiscriminate mixing of all proprioceptors; rather, some proprioceptors appear to “skip” the domains in their own segment of neuropil and form outputs only in adjacent segments. The limited number of output combinations and specificity in how neurons converge across segments may indicate combinations of feedback signals that are important to integrate into downstream circuits (Levinsson et al., 2002; Willis and Coggeshall, 2004). For instance, in the case of the central domain, convergence may create an area where locomotor-related proprioceptive feedback is integrated. Outputs in this domain likely encode information related to contraction of the current segment (dmd1), as well as information about the movement of either of the adjacent boundaries: movement forward of the anterior boundary (ddaE, and plausibly vpda, from the anterior segment), or movement backward of the posterior boundary (ddaD from the posterior segment) (He et al., 2019; Vaadia et al., 2019). This information could signal that a locomotor wave is progressing from the segment in question into the next.

Using our current dataset, in which a large fraction of proprioceptors’ downstream targets have yet to be identified and reconstructed, we cannot currently confirm whether spatial convergence corresponds to shared downstream target neurons or, conversely, whether spatial divergence leads to unique downstream neurons. This lacuna leaves many interesting questions to be answered. For instance, how unique are the downstream target neurons that receive input from each distinct spatial domain? Is spatially divergent proprioceptive information kept separated at the level of these second-order interneurons, or instead combined by these neurons across spatial domains and/or hemisegments? A few examples have already been described of downstream interneurons that make their own outputs on both sides of the midline and/or across segment boundaries, including some of the Even-skipped Lateral neurons (Heckscher et al., 2015) and the A27h neuron (Fushiki et al., 2016), both of which contribute to coordinating (hemi-)segmental contractions during locomotion. Examples are also known of downstream interneurons, such as Jaam interneurons, that receive proprioceptive inputs from both the left and right side of a segment (Heckscher et al., 2015). Either type of second-order interneuron connectivity could quickly combine proprioceptive information that was spatially segregated at the level of sensory outputs. We note that, even in cases where downstream target neurons combine information across multiple spatial domains (such as the Jaam neurons), the local divergence/convergence of proprioceptive subtypes we describe may still be important for local dendritic computations (Clarac and Cattaert, 1996; Hao et al., 2009; Pouille et al., 2013; Wilson, 2013).

Thus, from the functional perspective, an important next step will be to identify and reconstruct all downstream targets of proprioceptive neurons. This will help elucidate the extent to which spatial convergence and divergence of proprioceptive synapses actually represent integration or distribution of proprioceptive signals, respectively. From the developmental perspective, an important next step will be to understand the genetic control of synapse placement, in cases both of convergence and divergence.

### Little pre-synaptic inhibition onto larval proprioceptors

Presynaptic inhibition has been repeatedly described across proprioceptive systems and is considered a near-universal feature of proprioceptive sensory processing (Rudomin and Schmidt, 1999; Tuthill and Azim, 2018). Presynaptic inhibition of proprioceptive feedback is thought to play many roles in motor control, including reducing the gain of proprioceptive signals, stabilizing reflexes, and preventing oscillations that could otherwise be caused by delayed feedback (Rudomin and Schmidt, 1999; Rossignol et al., 2006; Windhorst, 2007; Fink et al., 2014). Here, we found few inputs to proprioceptors alongside evidence that many inputs are excitatory rather than inhibitory (Figure 4). This suggests that larval proprioceptors are distinctive, likely being subject to little to no presynaptic inhibitory control. What aspects of *Drosophila* larval body or behavior could explain this? In contrast to most proprioceptive systems that have been studied in depth (e.g., adult fly), larvae lack limbs; they move primarily using peristaltic contractions of consecutive body segments (Heckscher et al., 2012). This difference in body form and locomotor strategy could lead to differences in the organization of locomotor circuits that obviate the need for reflex stabilization or phase-dependent gating. Indeed, the “mission accomplished” model proposed for the role of proprioceptive feedback in larval locomotion does not apparently depend on presynaptic inhibition (Hughes and Thomas, 2007; Pehlevan et al., 2016). Alternatively, gain control may be important for some aspects of proprioceptive processing but implemented without presynaptic inhibition: via properties of the sensory neurons themselves or of their interaction with the body that reduce synaptic transmission depending on the animal’s behavioral state or the neuron’s firing history (Tuthill and Azim, 2018; Dickerson et al., 2021). Future modeling and experiments are needed to determine what properties of proprioceptors, or properties of proprioceptive processing, differentiate this sensory modality in larvae from other somatosensory modalities.

## Conclusion

Altogether, our results provide the most comprehensive and detailed map to date of a proprioceptive system’s earliest stage of central organization. This map opens the door to developmental and functional studies that will ultimately elucidate the relationship between the anatomical and functional organization of proprioceptive networks, a relationship that is fundamental for understanding how proprioception contributes to larval motor control.

## Data availability statement

The original contributions presented in this study are included in the article/Supplementary material, further inquiries can be directed to the corresponding author.

## Author contributions

MG and EH contributed to the conception and design of the study. CW performed annotations in the connectome. MG performed the analysis and wrote the manuscript. All authors contributed to manuscript revision, read, and approved the submitted version.

## Footnotes

1
https://github.com/navis-org/pymaid

2
https://github.com/HeckscherLab/proprio-synapse-org

## Supplementary material

Supplementary Figure 1 Output synapses in the “central posterior” domain (see Figure 3), corresponding to the “central domain” of segment A2. Top, body wall diagram: dendritic “receptive field” of the contributing neurons. Two views plotted below; contributing neurons colored according to body wall diagram. Note contribution of synapses from neurons in both adjacent segments. Spatial position values along axes are given in nm.

Supplementary Figure 2 Distributions of A1 proprioceptor output synapses along left-right (L-R/mediolateral) axis, **top** panel; along dorsal-ventral (D-V) axis, middle panel; and along anterior-posterior (A-P) axis, **bottom** panel (Direction of axis relative to CNS shown in insets). **Left**-side proprioceptors in **left** set of 3 panels; **right**-side proprioceptors in **right** set of 3 panels. Each proprioceptor’s output distribution plotted as the proportion of outputs in 1 μm bins, colored according to legend. Midline plotted as vertical dashed line.

Supplementary Figure 3 Anterior-posterior distributions of output synapses from individual proprioceptors from segments T3, A1, and A2 (or T3-A3, ddaD, and vbd only). Each segment’s output distribution plotted as the proportion of outputs in 1 μm bins; segments colored according to legend. Direction of the A-P axis shown relative to CNS at bottom.

Supplementary Figure 4 **(A)** Top-down views of output synapses from all T3, A1, and A2 chordotonals (that have been annotated), colored according to diagram. All chordotonals combined, with proprioceptor output synapses plotted in gray for spatial context, in top panel. Individual chordotonals’ T3–A2 outputs, with other chordotonals’ outputs plotted in gray, in lower set of panels. **(B)** Anterior-posterior (A-P) distribution of output synapses from individual chordotonals from segments T3, A1, and A2. (Direction of axis relative to CNS shown at bottom). Each segment’s output distribution plotted as the proportion of outputs in 1 μm bins; segments colored according to legend.

Supplementary Figure 5 **(A)** Top-down views of output synapses from all T3, A1, and A2 md cIV neurons (that have been annotated), colored according to diagram. All md cIVs combined, with proprioceptor output synapses plotted in gray for spatial context, in top panel. Individual md cIVs’ T3-A2 outputs, with other md cIVs’ outputs plotted in gray, in lower set of panels. **(B)** Individual md cIV neuron’s output synapses, this time colored by side and segment identity, according to legend. Other md cIVs’ outputs plotted in gray. Note output synapses spanning multiple segments and sometimes crossing midline. **(C)** Anterior-posterior (A-P) distribution of output synapses from individual md cIV neurons from segments T3, A1, and A2. (Direction of axis relative to CNS shown in inset). Each segment’s output distribution plotted as the proportion of outputs in 1 μm bins; segments colored according to legend.

Supplementary Table 1 Summary CATMAID information for neurons used in this paper.

Supplementary Table 2 Input and output synapse counts for each proprioceptive neuron in segments T3, A1, and A2, on the left and right side. Where both inputs and outputs entries are 0, the neuron did not exist in the annotated dataset.

Supplementary Table 3 Input and output synapse counts for each chordotonal and class IV multidendritic neuron in segments T3, A1, and A2. Where both inputs and outputs entries are 0, the neuron did not exist in the annotated dataset.
