# Supplementary figures and images for: Distinctive features of the central synaptic organization of Drosophila larval proprioceptors
Source: Front Neural Circuits. 2023 Jul 26;17:1223334. doi: 10.3389/fncir.2023.1223334 (PMC10410283; doi:10.3389/fncir.2023.1223334)

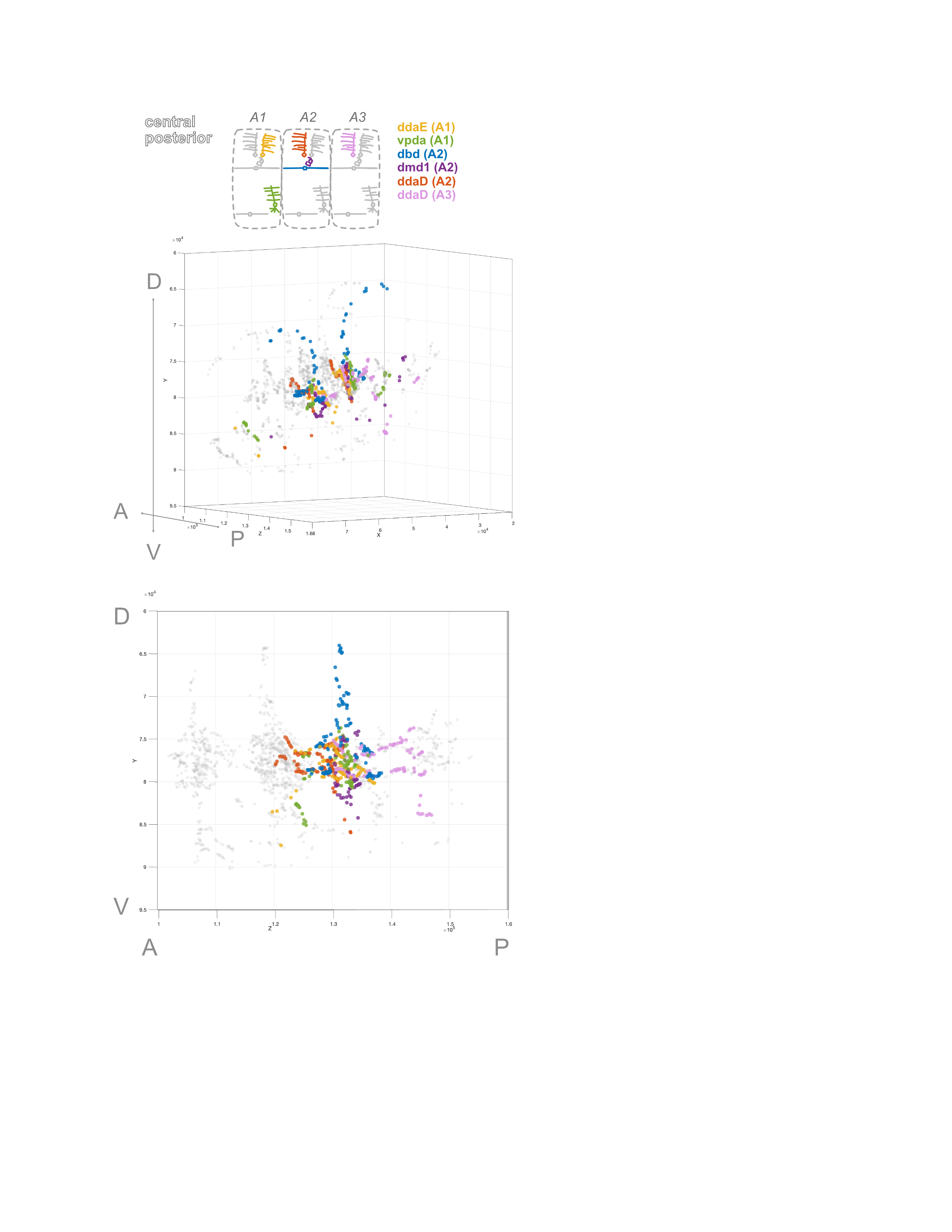

Supplement: Supplementary Figure 1 — Output synapses in the “central posterior” domain (see Figure 3), corresponding to the “central domain” of segment A2. Top, body wall diagram: dendritic “receptive field” of the contributing neurons. Two views plotted below; contributing neurons colored according to body wall diagram. Note contribution of synapses from neurons in both adjacent segments. Spatial position values along axes are given in nm. [file Image_1.TIFF]

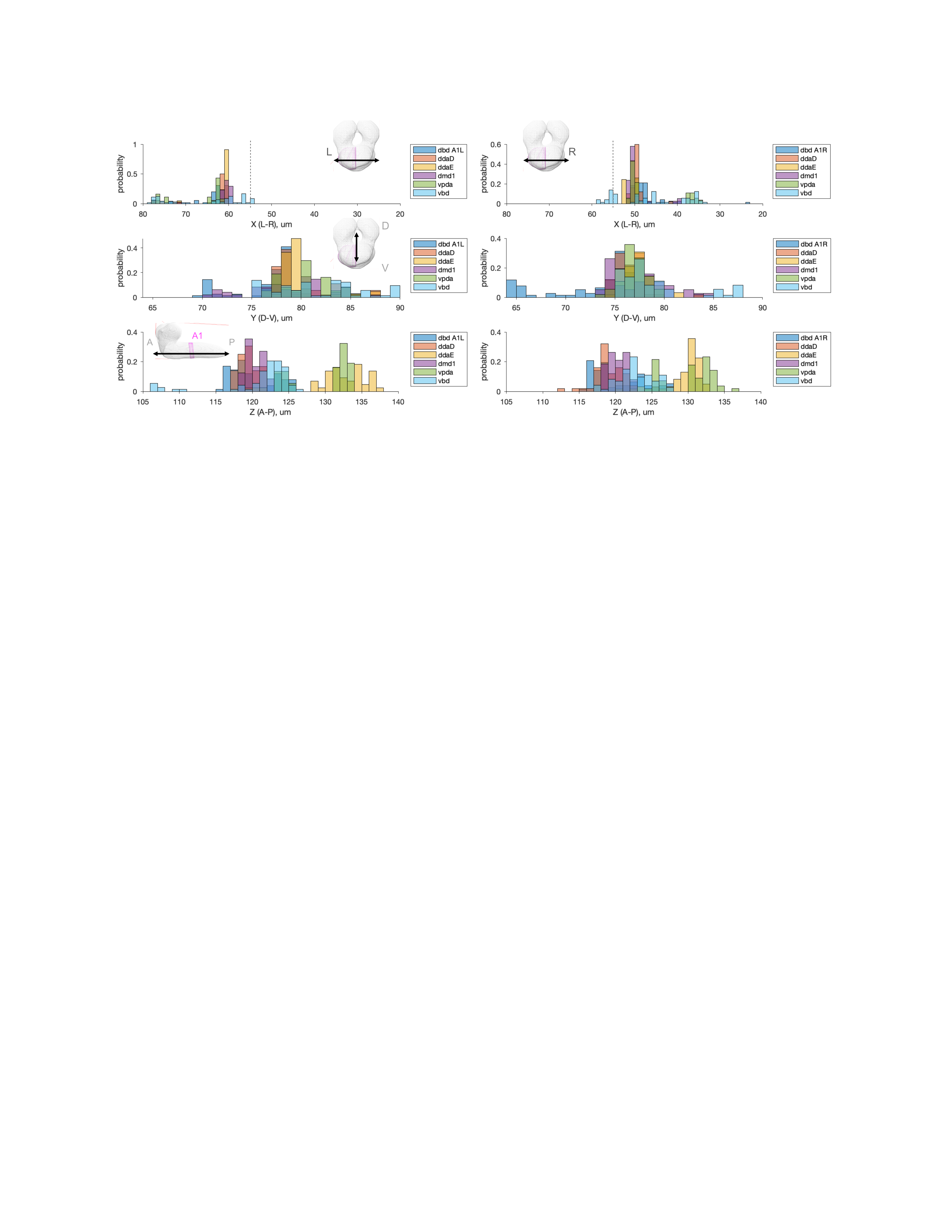

Supplement: Supplementary Figure 2 — Distributions of A1 proprioceptor output synapses along left-right (L-R/mediolateral) axis, top panel; along dorsal-ventral (D-V) axis, middle panel; and along anterior-posterior (A-P) axis, bottom panel (Direction of axis relative to CNS shown in insets). Left-side proprioceptors in left set of 3 panels; right-side proprioceptors in right set of 3 panels. Each proprioceptor’s output distribution plotted as the proportion of outputs in 1 μm bins, colored according to legend. Midline plotted as vertical dashed line. [file Image_2.TIFF]

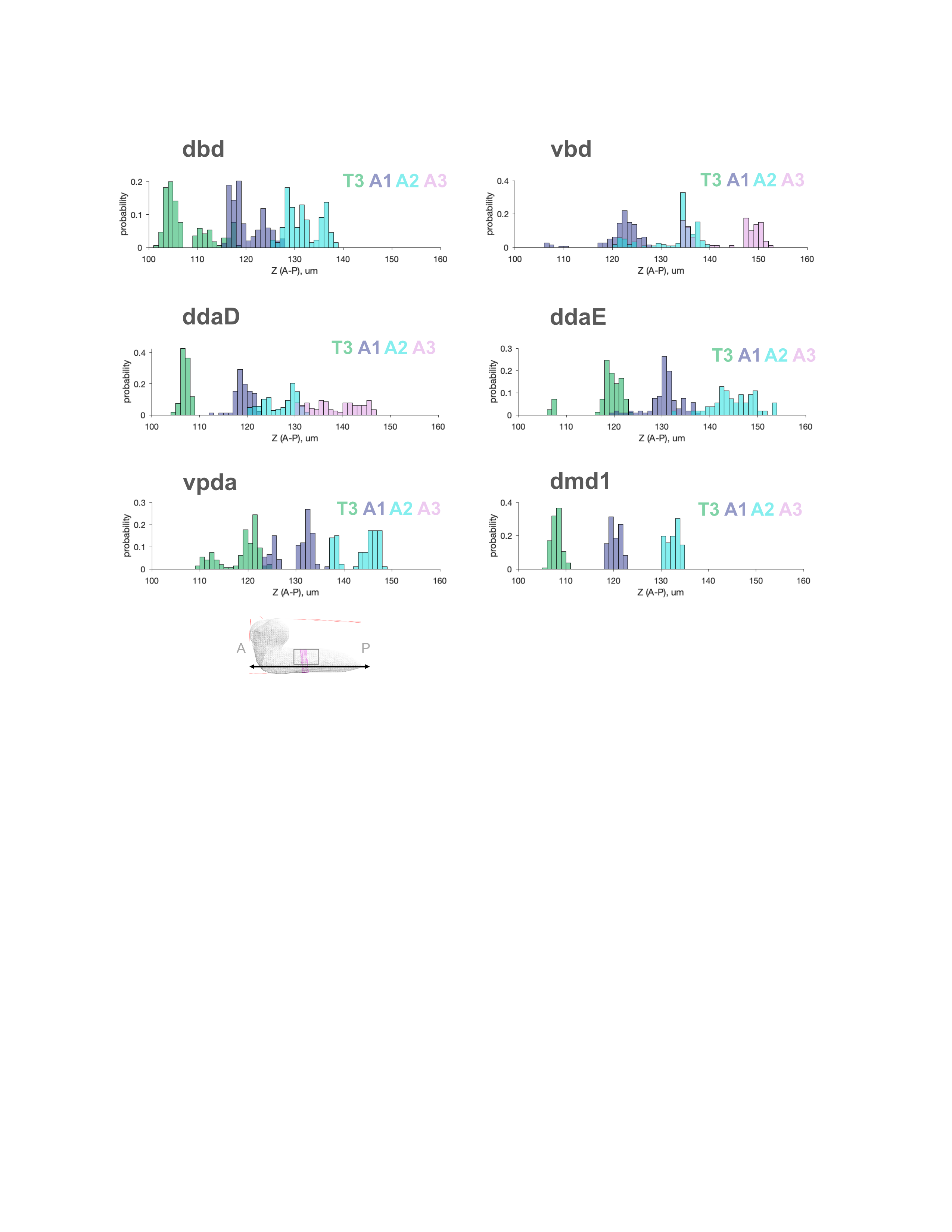

Supplement: Supplementary Figure 3 — Anterior-posterior distributions of output synapses from individual proprioceptors from segments T3, A1, and A2 (or T3-A3, ddaD, and vbd only). Each segment’s output distribution plotted as the proportion of outputs in 1 μm bins; segments colored according to legend. Direction of the A-P axis shown relative to CNS at bottom. [file Image_3.TIFF]

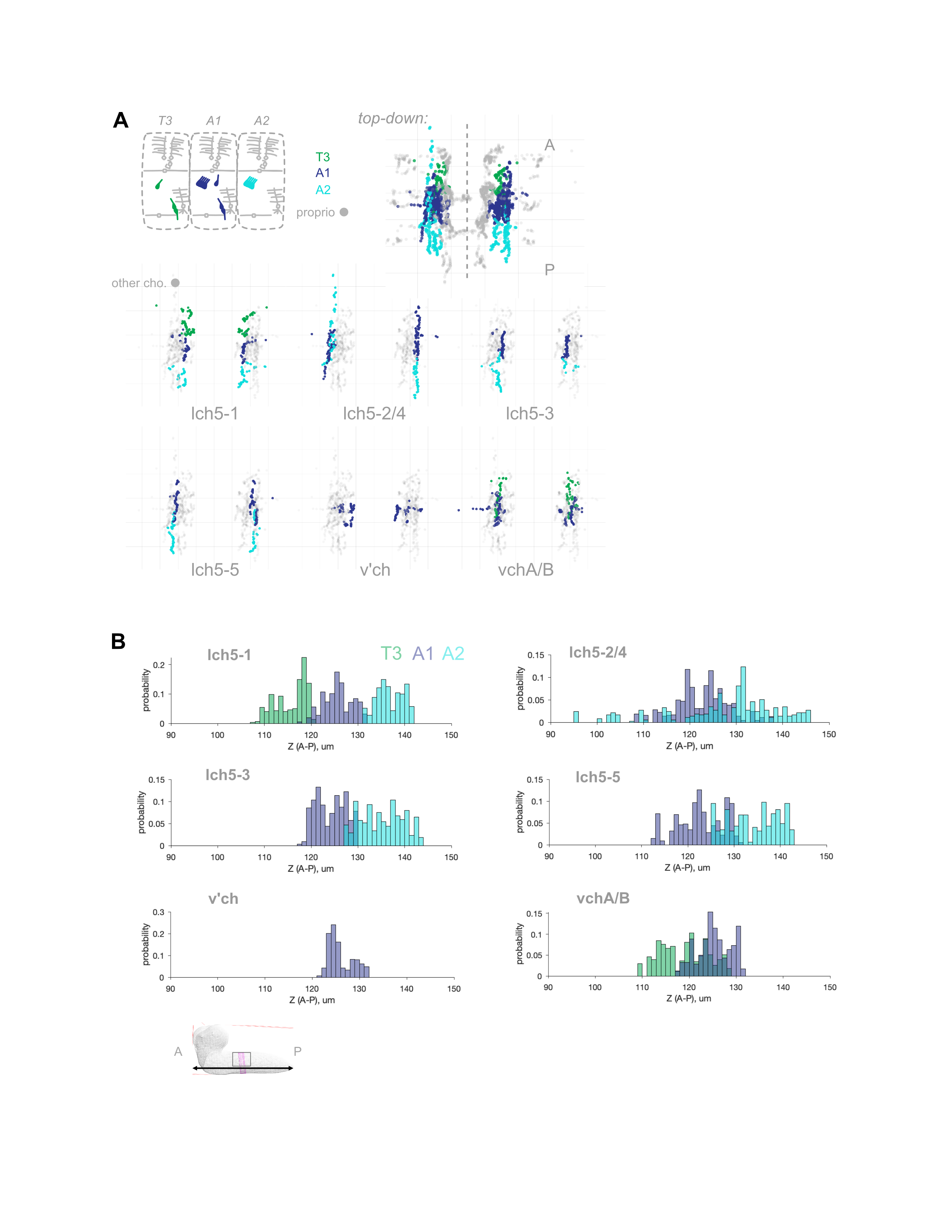

Supplement: Supplementary Figure 4 — (A) Top-down views of output synapses from all T3, A1, and A2 chordotonals (that have been annotated), colored according to diagram. All chordotonals combined, with proprioceptor output synapses plotted in gray for spatial context, in top panel. Individual chordotonals’ T3–A2 outputs, with other chordotonals’ outputs plotted in gray, in lower set of panels. (B) Anterior-posterior (A-P) distribution of output synapses from individual chordotonals from segments T3, A1, and A2. (Direction of axis relative to CNS shown at bottom). Each segment’s output distribution plotted as the proportion of outputs in 1 μm bins; segments colored according to legend. [file Image_4.TIFF]

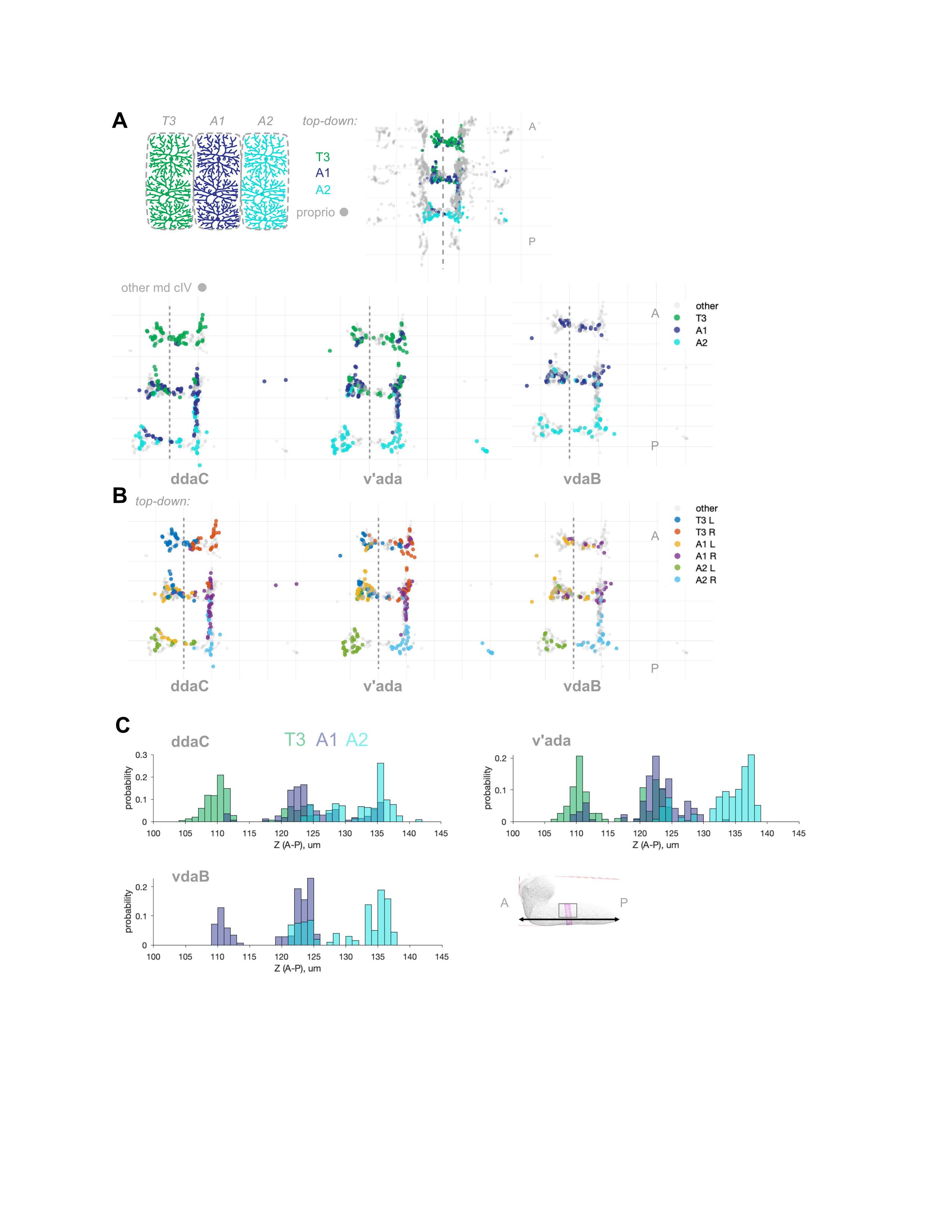

Supplement: Supplementary Figure 5 — (A) Top-down views of output synapses from all T3, A1, and A2 md cIV neurons (that have been annotated), colored according to diagram. All md cIVs combined, with proprioceptor output synapses plotted in gray for spatial context, in top panel. Individual md cIVs’ T3-A2 outputs, with other md cIVs’ outputs plotted in gray, in lower set of panels. (B) Individual md cIV neuron’s output synapses, this time colored by side and segment identity, according to legend. Other md cIVs’ outputs plotted in gray. Note output synapses spanning multiple segments and sometimes crossing midline. (C) Anterior-posterior (A-P) distribution of output synapses from individual md cIV neurons from segments T3, A1, and A2. (Direction of axis relative to CNS shown in inset). Each segment’s output distribution plotted as the proportion of outputs in 1 μm bins; segments colored according to legend. [file Image_5.tiff]
